# Impact of Low Volume Velocity-Controlled vs. Repetition to Failure Resistance Training Session on Measures of Explosive Performance in a Team of Adolescents Basketball Players

**DOI:** 10.3390/sports9080115

**Published:** 2021-08-23

**Authors:** Ott-Erik Kalmus, Mehis Viru, Brent Alvar, Fernando Naclerio

**Affiliations:** 1 Centre for Exercise Activity and Rehabilitation, Institute for Lifecourse Development, School of Human Sciences, University of Greenwich, London SE10 9LS, UK; F.J.Naclerio@greenwich.ac.uk; 2 Institute of Sports Sciences and Physiotherapy, Faculty of Medicine, University of Tartu, 51008 Tartu, Estonia; mehis.viru@ut.ee; 3 Department of Kinesiology, Point Loma Nazarene University, San Diego, CA 92106, USA; balvar@pointloma.edu

**Keywords:** velocity-based training, post-activation potentiation, team-sport, vertical jump

## Abstract

This study examined the short-term effects (post 6 h and 24 h) of two equated (70% of 1 repetition maximum (1-RM)) low volume resistance exercise protocols: (i) velocity-controlled (VC) and (ii) repetition to failure (RTF) on upper and lower body performance in competitive adolescent male basketball players. Following a randomized, counterbalanced design, ten participants (age: 16 ± 0.5 years) completed either VC or RTF separated by 72 h. VC consisted of 4 sets of 5 explosive repetitions (≥90% of the maximum velocity). RTF involved 2 sets of 10-RM (with no velocity control). Measurements of 20-m sprint, countermovement jump (CMJ) and medicine ball toss (MBT) were collected before (baseline), post 6 h and 24 h after either VC or RTF. Increases of CMJ post 6 h (VC, +6.7%; RTF, +2.4%) and MBT post 24 h (VC, +4.6%; RTF, +4.2%) were observed after both VC and RTF. Only VC potentiated CMJ after 24 h (+2.0 ± 2.3%). No other changes or differences between protocols were observed. Performing a low volume exercise protocol, either VC or RTF, induced similar potentiation effects on the vertical jump (post 6 h) and medicine ball toss (post 24 h) in adolescent basketball players. Only the VC protocol was still effective to potentiate CMJ performance after 24 h.

## 1. Introduction

Post activation potentiation (PAP) is a physiological neuromuscular capacity inherent in all individuals regardless of performance level and training experience [1]. PAP is a concept used by strength and conditioning coaches and athletes to strategically increase power output during short time periods by preloading the targeted muscles and/or actions through exercise selection [2]. Previous studies have reported such potentiation enhancement effects on impulsive strength capacities after the completion of high intensity, low volume resistance training workouts [2,3,4]. Initially, the PAP effect was associated with an enhanced muscle contractile response for a given level of stimulation following an intense voluntary contraction, which is measured as the maximum twitch force evoked by supramaximal electrical stimulation [5]. This effect can be explained by an increased recruitment of higher-order motor units along with the rate of phosphorylation of myosin regulatory light chains [5,6], which can elicit potentiation for a subsequent short period of time (<5 min) [7]. Nonetheless, the time course of the previously described classic PAP effect is shorter than the reported window of potentiation observed in several studies (>3 to 16 min) [8]. Consequently, given the different time course of effect along with possible differences in the mechanisms underpinning the performance enhancement effect, the term “post-activation performance enhancement” (PAPE) is proposed [9]. Therefore, PAPE occurs when a high-intensity voluntary conditioning exercise is performed with the aim to enhance a subsequent voluntary muscular action rather than it being electrically evoked (twitch) [9,10,11]. The PAPE effect could potentially be achieved via mechanisms such as increased body temperature and increased neural drive or water content [11]. Despite not knowing the exact mechanisms underpinning PAPE, it can be achieved by using various external loads and training volume, even eliciting the performance enhancement effect several hours after having performed the conditioning activity [11]. For instance, Saez de Villareal et al. [3] reported a significant increase of vertical jump performance measured at both 5 min and 6 h after performing a squat conditioning protocol using 80–95% of 1-RM. West et al. [12] observed improved peak power output measured 8 min after completing a low volume bench press protocol either using low (30% of 1-RM) or high loads (87% of 1-RM). The short latency period between preconditioning and the subsequent explosive exercises is usually between one to five minutes (PAP), while the existence of a long latency period (PAPE) of up to 6 h has also been identified [4,11,12,13]. The ideal recovery time between conditioning exercises and post activation performance enhancement responses are not well understood and seem to be influenced by individual factors [14]. Nonetheless, the use of PAP or PAPE continues to be a popular strategy despite the potential physiological attributes for increasing power-output in subsequent impulsive vertical [15] or long jump exercises [16]. In addition, it can be useful for team sport athletes, who usually have a reduced time over competitive microcycles, to integrate strength and conditioning workouts and competitions.

A recently proposed velocity-based training (VBT) method [17] has been shown to contribute to both muscular and neural mechanisms favoring power-output and strength adaptations in athletes [18]. During VBT, athletes train with a target range (e.g., >90% to maximal) of movement velocity. Velocity loss (VL) thresholds are calculated from the initial repetitions in order to terminate the set when movement velocity drops to a certain percentage (e.g., a 10% VL). Using lower VL (e.g., 10%) during resistance exercises has proven to be effective to minimize fatigue and increase or maintain an average power peak or vertical velocity across the entire workout session [19]. As such, it is possible to apply VBT principles to limit fatigue during pre-conditioning exercises (e.g., squat or bench press) to subsequently enhance specific neuromuscular performance (e.g., vertical jump or medicine ball throw). Without the use of VBT principles, training outcomes could potentially shift from power towards strength or muscular endurance by training to failure or near failure. This type of training yields a significant drop in movement velocity, which can be avoided by tracking bar speed [16,19,20].

To the best of the authors’ knowledge, no study has previously compared the effects of a low volume VBT vs. a traditional repetition to failure protocol on sport-specific performance measured after 6 and 24 h. As the implementation of different training methods could provide coaches with an insight on how to develop protocols to increase short-term high impulse performance, this study aimed to examine the effects of two low volume-equated RT protocols: (i) velocity-controlled (VC) and (ii) repetition to failure (RTF) on lower and upper body explosive performance measured after 6 and 24 h of completing either a VC or an RTF protocol in adolescent male basketball players. Based on the available literature it is hypothesized that a low volume VC routine will elicit performance enhancement after both 6 and 24 h while no change will be observed following the implementation of the RTF protocol.

## 2. Materials and Methods

### 2.1. Experimental Design

**Figure 1:**
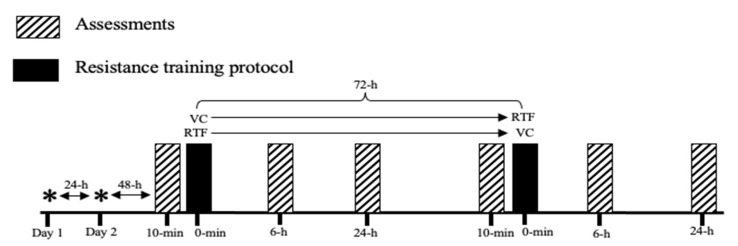
Schematic representation of the study. Assessments are conducted at baseline (test 0), 6 h (test 1) and 24 h (test 3) after the completion of either velocity control or repetition to failure protocols. Day 1 *: familiarization; Day 2 *: anthropometrics measurements and 5-RM testing, VC: velocity-controlled; RTF: repetition to failure.

The current study used a randomized repeated measure counterbalanced design, where participants served as their own controls. Following a familiarization period, participants performed (i) a 20-m sprint (S-20), (ii) a countermovement jump (CMJ), and (iii) a seated 4 kg medicine ball toss (MBT) as measures of performance, at baseline (before), 6 h and 24 h after the completion of two different low volume-equated resistance training protocols (VC and RTF) composed by two lower-body (parallel squat and hip thrust) and one upper-body (bench press) exercise (Figure 1).

### 2.2. Participants

Ten male adolescent basketball players (age 16 ± 0.5 years, height 189.4 ± 5.4 cm, body mass 79 ± 16 kg) with a minimum of two years of participation in regular structured resistance training and competing for the same team in the U18 Championship League volunteered to participate in this study. The recruited sample represented all the possible candidate matching with the inclusion criteria for the present study.

Written informed consent was obtained from all participants and participants’ parents/guardians before the start of the study. All participants and parents/guardians were notified of the research procedures, protocols, benefits, and risks before providing written consent. A health history questionnaire was used to ensure that participants were healthy and free of any musculoskeletal injury or cardiovascular disease. Only individuals non-ingesting ergogenic aids or any type of nutritional supplements affecting muscular performance for 12 weeks or longer before the start of the study were recruited. The study was approved by the institutional ethics committee at the university and carried out in accordance with the guidelines contained in the Declaration of Helsinki.

### 2.3. Procedures

To avoid interference with competitions and to facilitate compliance with the study procedures, all the participants were assessed after the end of the competition period (off-season). Participants reported to the laboratory on 8 separate occasions. To ensure the proper understanding of exercises and testing procedures, the first session was allocated for familiarization purposes. After 24 h (2nd session), measurement of body mass and height were collected using a standard scale and a stadiometer [21]. Thereafter, within the same testing session, a five-repetition maximum test (5-RM) was conducted for each of the three conditioning exercises: parallel back squat, bench press and hip thrust. An 8-min recovery period was allowed between tests. For the 5-RM test, subjects initially performed 5 repetitions with 50% of the estimated 1-RM. Load increments were conducted based on participants’ perception of effort until reaching the 5-RM load [22]. Eventually, the highest load possible to lift in one maximum repetition (1-RM) was estimated using the Epley equation [22].

The two resistance exercise protocols were performed 72 h apart (sessions 3 and 6). Both were followed by two assessment sessions, conducted 6 h (sessions 4 and 7) and 24 h (sessions 5 and 8) after completing either the VC or RTF protocols (Figure 1). To minimize any diurnal effects on performance, the assessment time was constant throughout the study period. Baseline assessments were conducted between 9 to 11 a.m. about 10 min before performing either the VC or RTF conditioning workouts. The post-workout assessments were conducted between 5 to 6 p.m. and between 11 to 12 a.m., after 6 h and 24 h, respectively, of completing the corresponding conditioning workout. To standardize recovery procedures over 24 h post exercise, under the two analyzed conditions and for the entire intervention, participants were instructed not to change their nutritional habits, and if relevant modifications were reported (i.e., becoming vegetarian, restricting calories), participants’ data were excluded from the analysis. Additionally, the informed consent indicated the need to sleep at least 7 to a maximum of 9 h the night before testing, and subjects were allowed to drink water ad libitum.

Before testing and training, participants carried out a standardized warm-up involving light dynamic calisthenics exercises, one set of 5 to 8 repetitions for each of the three training exercises using a self-selected load ~50% of 1-RM, one short sprint (~15 m), two MBTs and two CMJs. Thereafter, participants completed a baseline assessment followed by a 10-min recovery period and underwent either the VC or RTF resistance exercise protocol.

#### 2.3.1. Assessments

Linear 20-m sprint (S-20). Three S-20 sprint times were recorded using two infrared photoelectric cells (Newtest Powertimes, Finland). The starting line was placed 30 cm before the first infrared cell and the sprint start position was used at all times. The participants performed three attempts, separated by 3 min of recovery. The best performance (fastest sprint) of three attempts was selected for the analysis.

Countermovement jump (CMJ). From a standing erect position, participants descended to a self-selected depth and immediately jumped upward as high as possible. To exclude the influence of arm-swing, participants were instructed to keep their hands on the hips. At least 30 s of rest was provided between jumps. The best performance (highest value) of three attempts was considered for the analysis. An infrared portable mat device IVAR (IVAR, Estonia) was used to record flight times and thereafter calculate jump heights [23].

Seated 4 kg medicine ball toss (MBT). Three seated 4-kg medicine ball tosses were performed using the methodology described by Viitasalo [24]. Based on the distance, the best of three attempts was chosen for the analysis. A 30-s rest was allowed between attempts.

#### 2.3.2. Training Exercises

Parallel Back Squat. Using a squat rack, the bar was centered across the shoulders just below the spinous process of the C7 vertebra (high-bar position) [25]. From a standing position with feet parallel, shoulder width apart and toes pointing slightly outward, the participants were instructed to squat down with a controlled velocity until their posterior thigh was positioned parallel to the floor. After a minimum pause (less than 1 s), participants performed the concentric squatting phase with the maximal possible velocity.

Bench Press. The participants started lying down on a bench with their elbows fully extended to begin lowering the bar towards the chest using a controlled velocity [26]. After a minimum pause (less than 1 s), participants performed the concentric phase with the maximal possible velocity.

Barbell Hip Thrust. The participants started with their scapula as the pivot point on the bench (45 cm from the floor), feet slightly wider than hip-width and toes pointed forward with the bar placed over their lower abdominal and pubic region [27]. The eccentric portion of the lift was controlled until the barbell nearly contact the floor. Thereafter, the participants explosively extended the hips, raising the bar as fast as possible, ending the movement with the torso parallel to the floor and a neutral hip position.

#### 2.3.3. Conditioning Resistance Exercise Protocols

Either VC or RTF protocols involved the execution of the three previously described exercises performed in the same order, equated by the relative load (~70% 1-RM), the total number of repetitions per exercises and routine. In both protocols, the participants were instructed to perform the concentric phase of the 3 exercises with their maximal movement velocity at all times (Table 1).

VC involved the execution of 4 sets of 5 repetitions with a 2-min rest between sets per exercise. A linear position transducer (Speed4Lift, Madrid, Spain) was used to measure the mean concentric movement velocity. The validity and reliability (coefficient of variation = 2.61%) of the used device was reported previously [28]. The processing unit was placed on the floor just under the bar. A retractable cable was attached on the right side of the barbell to allow a vertical displacement of the bar over the complete range of motion for the three used exercises. The system automatically calculated kinematic measurements of every repetition and provided visual and auditory feedback to facilitate the control of velocity and the corresponding threshold. The participants were required to complete all repetitions at or above 90% of the maximum vertical velocity measured at the beginning of the first set (≤10% velocity loss threshold) [20]. If a participant was unable to maintain the requested movement velocity, the set was stopped by an auditory signal and the load was decreased by 5%. Thereafter, the participants were allowed to complete the total prescribed number of repetitions [20]. In order to promote maximal intent during the concentric portion of the exercises, verbal feedback regarding the achieved movement velocity (e.g., differences to the threshold limit) was provided after the completion of each singular repetition [29].

RTF involved 2 sets of 10 at ~70% 1-RM with a 3-min rest. The OMNI-RES scale (0–10) was used to adjust the load [30]. An initial OMNI-RES value of >4 and <6 was recommended for starting each set while a score of 10 was expected after the completion of the 10th repetition [31]. When participants could not reach the desired number of repetitions, an additional 15 s of rest was allowed until the total number of prescribed repetitions was completed for every set. Conversely, when the rating of perceived exertion (RPE) was lower than 10 after completing the 10th rep, the load was increased by 1.25 to 2.5 kg for the subsequent sets. A large (A3 size 29.7 × 42 cm) figure showing the OMNI–RES (0–10) scale was in full view of participants during the parallel squat (on the wall, less than 2 m in front) and bench press or hip thrust (held by a researcher, about 1 m above).

One qualified instructor (a certified strength and conditioning coach, CSCS) monitored the appropriate range of motion and correct execution of the three training exercises.

### 2.4. Statistical Analysis

A descriptive analysis was performed, and subsequently, the Shapiro-Francia tests were applied to assess normality. Before testing the main hypothesis, the possible treatment order effect was checked. For all the analyzed variables, a preliminary test using the sum of all values obtained for each participant at both conditioning training protocols (VC and RTF) was calculated and compared across the two sequenced conditions (VC-RTF vs. RTF-VC). An independent sample Student’s t-test was performed to compare the values measured in the five participants who started with VC vs. the results determined for the five others who started with RTF [32]. A two-way repeated measure analysis of variance (ANOVA) was used to examine differences between conditions (VC and RTF) and times (baseline, post 6 h and post 24 h) for each of the analyzed variables (S-20, CMJ and MBT). Bonferroni-adjusted pairwise comparison was used to compare differences between conditions and times when appropriate. Difference to baseline was also assessed as a percentage based on the equation: (% change = max/baseline × 100), where max is the best value for each protocol and baseline serves as the 100% mark. Eta squared (η2) and Cohen’s d values were reported to provide an estimate of the standardized effect size (small η2 = 0.01, *d* = 0.2; moderate η2 = 0.06, *d* = 0.5; and large η2 = 0.14, *d* = 0.8). All results are reported as mean (standard deviation) and 95% confidence intervals (CI) unless stated otherwise. The significance level was set at 0.05. All analyses were performed using SPSS for Windows, version 20.0 (SPSS, Inc., Chicago, IL, USA).

## 3. Results

Ten participants completed the intervention with 100% compliance. The mean ± SD in the 1-RM parallel squat, bench press and hip thrust was 123.0 ± 20.0 kg (1.55 ± 0.18 kg·kg^−1^ BM^−1^), 70 ± 12.5 kg (0.87 ± 0.12 kg·kg^−1^ BM^−1^) and 177.0 ± 39.0 kg (2.21± 0.39 kg·kg^−1.^ BM^−1^), respectively. No carryover effect was observed for S-20, CMJ and MBT variables (all *p* > 0.05).

### 3.1. Dependent Variables

No differences between conditions were determined at baseline for the three analyzed outcome measures (S-20, CMJ and MBT, all *p* > 0.05).

### 3.2. Linear 20-m Sprint (S-20)

No effect for condition (F_(1,9)_ = 2.172, *p* = 0.17, $\eta_{G}^{2}$ = 0.194), time (F_(2,18)_ = 2.293, *p* = 0.13, $\eta_{G}^{2}$ = 0.203) and interaction (F_(2,18)_ = 0.581, *p* = 0.57, $\eta_{G}^{2}$ = 0.061) was determined.

### 3.3. Vertical Jump (VJ)

**Figure 2:**
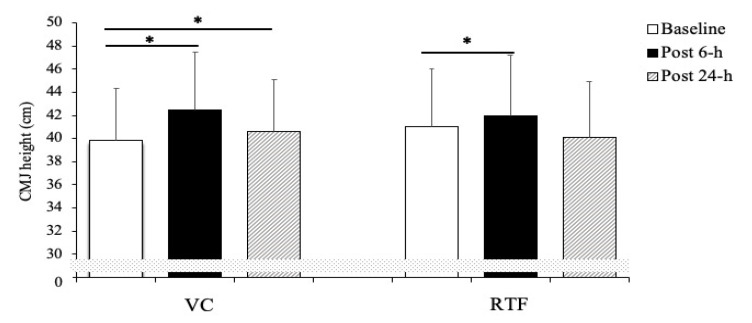
Comparison between baseline and post conditions countermovement jump (CMJ) height (mean and 95% CIs) performance, determined either for velocity-controlled and repetition to failure condition regardless of the time point. VC: velocity control, RTF: repetition to failure. * *p* < 0.05 compared to baseline.

Even though no main effect for condition (F_(1,9)_ = 0.047, *p* = 0.83, $\eta_{G}^{2}$ = 0.005) was observed, main effects for time (F_(2,18)_ = 15.112, *p* = 0.01, $\eta_{G}^{2}$ = 0.627) and interaction (F_(2,18)_ = 4.153, *p* = 0.03, $\eta_{G}^{2}$ = 0.316) were determined. The post-hoc analysis revealed significant performance enhancement effects after 6 h for both conditions (VC, *p* = 0.001 *d* = 1.53; RTF, *p* = 0.05 *d* = 0.81), while after 24 h only the VC condition significantly enhanced vertical jump (VJ) height (*p* = 0.032 *d* = 0.80). No other differences to baseline or between conditions were identified. Figure 2 describes the mean values and 95% CIs estimated at the three time points for both VC and RTF condition.

After performing the VC protocol, the participants improved VJ performance by 6.7 ± 4.3% and 2.0 ± 2.3% after 6 and 24 h, respectively. Conversely, after the completion of the RTF workout a 2.4 ± 3.7% increase was observed after 6 h while a decrease of 2.1 ± 5.4% was observed at 24 h.

### 3.4. Seated 4 kg Medicine Ball Toss (MBT)

**Figure 3:**
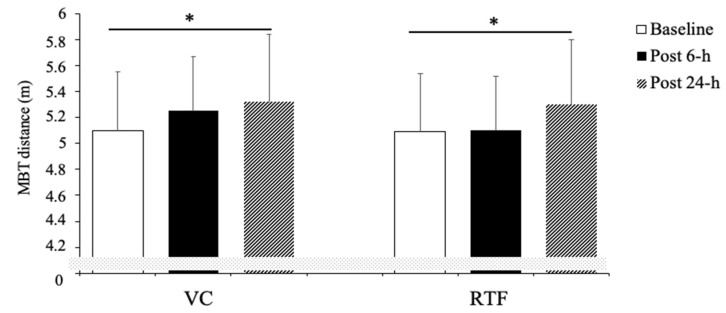
Comparison between baseline and post condition seated 4 kg medicine ball toss (MBT) distance (mean and 95% CIs) performance, determined either for velocity-controlled and repetition to failure condition regardless of the time point. VC: velocity control, RTF: repetition to failure. * *p* < 0.05 compared to baseline.

A significant main effect for time (F_(2,18)_ = 7.203, *p* = 0.01, $\eta_{G}^{2}$ = 0.445) was observed. However, no effects for condition (F_(1,9)_ = 0.412, *p* = 0.54, $\eta_{G}^{2}$ = 0.044) or interaction (F_(2,18)_ = 0.664, *p* = 0.53, $\eta_{G}^{2}\;$= 0.069) were determined. The post-hoc analysis revealed significant performance enhancement for the MBT for both conditions after 24 h (VC *p* = 0.015 *d* = 0.95, RTF *p* = 0.03 *d* = 0.81). No other differences to baseline or between conditions were identified. Figure 3 describes the mean values and 95% CIs estimated at the three time points for both VC and RTF condition.

After performing the VC protocol, the participants improved MBT by 3.3 ± 4.7% and 4.6 ± 4.5% after 6 and 24 h, respectively. Similarly, after completion of the RTF workout the distance achieved in the MBT increased by 1.2 ± 3.6% and 4.2 ± 4.6% at 6 h and 24 h, respectively.

## 4. Discussion

Results of the present study suggest that performing a low volume resistance training protocol using either velocity-controlled or repetitions to failure conditions 6 h to 24 h prior to performing short sprints and impulsive upper and lower body exercises causes no detrimental effect on performance. Additionally, both protocols promoted increments on jumping (post 6 h) and throwing capacity (post 24 h). Furthermore, the VC condition seems to be appropriate to benefit jumping abilities for up to 24 h after the completion of a low volume workout session. Based on these findings, and within the confines of the study procedures, we can only partially accept our research hypothesis asserting that the low VC routine will elicit performance enhancement at both 6 and 24 h regarding improving VJ and after 24 h regarding improving MBT. Additionally, we confirm the lack of PAPE effects elicited by the low volume RTF protocol only after 24 h for the CMJ and 6 h for the MBT after having completed the conditioning protocol.

Several studies examined neuromuscular performance after resistance exercises followed by short recovery periods [11,13]. Nonetheless, to the best of the authors’ knowledge, only a few investigations have analyzed and compared the effect of different low volume resistance exercise protocols on subsequent impulsive performance measured after 6 and 24 h [3,33].

In line with our findings, Saez de Villarreal et al. [3] observed no performance enhancement effects on jumping performance after 6 h of having completed a low volume protocol using 80–90% of 1-RM in well-trained volleyball players. As observed in our study, jumping ability was maintained, with no further performance impairment over 24 h. Current literature suggests that the enhancement effect occurs at some point over a window of potentiation lasting for a few seconds up to 12 min [14] or 16 min [8] after performing different volumes of conditioning activity. Indeed, in line with our results, Tsoukos et al. [33] observed a 3–5% increase in CMJ performance 24 and 48 h after performing multiple sets of squat jumps using 40% of the 1-RM in squat. The relative load used by Tsoukos and colleagues was lower than the load used in the current study and therefore elicited lower levels of fatigue than what was observed in the current study’s participants, particularly when performing the RTF condition. In addition, exercising with maximal movement velocities (e.g., >90% of maximal velocity) has proven to help avoid metabolic fatigue [19]. Moreover, near or maximal execution velocity may favor selective fast motor unit recruitment [18], while the intention to complete the exercises with the highest possible intensity has shown some potentiation effects by acutely improving subsequent explosive performance [12,16]. The current study evaluated post activation performance enhancement over 6 h and 24 h, where the induced calcium sensitivity via an enhanced myosin light chain phosphorylation will have been dissipated [34]. However, neural factors could result in PAPE through an increased synaptic efficacy between afferent terminals and α-motoneurons, among other mechanisms such as body temperature or water content, which have been associated with a delayed potentiation effect [9].

Cook et al. [35] observed increases in mechanical power output and maximal sprinting abilities after performing either a strength or a sprint training session, respectively. The authors suggested that sprint times were improved only when sprints were performed in the morning session and mechanical power improved only when resistance training was conducted as a preconditioning activity in the morning session. Our results showed no detrimental or performance enhancement effect on the 20-m sprint. The lack of a sprinting-specific conditioning exercise could have been the cause of the observed response. Similar results have been reported by Russel et al. (2015), who observed improvements in lower body explosive actions (sprinting and jumping) performed during the afternoon after the completion of repeated sprints “priming” exercises during a morning training session [10].

On the other hand, Cook et al. [34] suggested that performance increases resulted from PAP or PAPE could also be related to increased motivation. Participants of the current study received auditory feedback regarding the movement velocity after each repetition during the VC protocol. By providing real-time feedback of movement velocity, an increase in participants’ motivation and thus improved performance may have occurred [20]. To minimize the influence of other non-neuro-physiological factors associated with the PAP phenomenon, previous studies have considered minimum threshold changes from baseline to identify meaningful potentiation effects [14]. Accordingly, regardless of the absolute differences, any change ≥5% from baseline can either be considered as a positive or negative effect on the subsequent performance, whereas all performances within this range (<5% change) were considered as no potentiation or no detrimental response [13]. Interestingly, PAP is a physiological neuromuscular capacity producing different degrees of potentiation resulting from interindividual variability [13]. Therefore, participants, who increased their performance by more than 5% can be classified as responders, while those with no potentiation (changes <5% from the baseline measure) could be considered as non-responders, at least for the window of time tested in each particular study. Based on this rationale, it seems that both conditioning protocols, VC and RTF, elicited no potentiation for any of the dependent variables explored in the present investigation (S-20, CMJ and MBT). Nonetheless, analyzing the participants’ individual responses, seven out of ten improved their CMJ performance by ≥ 5% at 6 h and two at 24 h when exercising after completing the VC condition. Conversely, after performing the RTF condition, only two participants improved CMJ performance at 6 h and one participant after 24 h. Furthermore, two participants showed a meaningful performance decrease of ≥5% at 6 h and 4 participants at 24 h. The previous analysis advocates for the use of VC over the RTF condition to enhance subsequent (post 6 h and 24 h) neuromuscular performance.

Our participants performed two low volume resistance exercise protocols using similar relative moderate loads (~70% of 1-RM) and volume (total number of repetitions) but following two different set configurations and rest periods. The VC condition was designed to produce a high level of neuromuscular activation with no metabolic fatigue [36]. Conversely, the RTF condition was intended to cause a considerable level of metabolic and central fatigue. Research indicates that exercising to failure can cause significant muscle damage and long-lasting peripheral fatigue with a concomitant higher detrimental effect on the assessed performance outcome [37]. However, the lack of differences in the neuromuscular performance measured under VC vs. RTF conditions seems to support the notion that a low volume resistance session can be conducted without a detrimental effect on subsequent (6 to 24 h) sprint or upper and lower body impulsive performance. Therefore, including low volume strength training aimed at maintaining power related outcomes, prior to competitions or heavy training, appear to be an acceptable strategy for team sport athletes. Strength and conditioning coaches may want to consider implementing low volume exercise protocols composed of three exercises performed either with VC (e.g., 4 sets of 5 reps moved with maximal velocity and 2 min rest) or RTF (e.g., 2 sets of 10-RM with 3 min rest) expecting no detrimental effect or even to induce some performance improvements after 6 h or 24 h of having completed the workout sessions.

## 5. Conclusions

In conclusion, performing a low volume resistance workout lifting with moderate loads (~70%) either explosively (≥90% of the maximum velocity) for a low number of repetitions per set or reaching the momentary muscle failure per set (10-RM) did not cause any detrimental effect on 20-m sprint, jumping or throwing performance measured at 6 h and 24 h post training. However, significant performance enhancement was observed under both conditions at 6 h (jumping) and 24 h (throwing) of having completed the assigned low volume resistance exercise routine. Improvement of jumping performance after 24 h was produced only after performing the velocity-controlled not the repetition to failure protocol.

## Figures and Tables

**Table 1:** Summary of conditioning resistance training protocols.

Variable	Velocity Control Protocol	Repetitions to Failure Protocols
Sets × reps	4 × 5	2 × 10
Load	70% of 1-RM	70% of 1-RM ^1^
Rest period	2 min	3 min
Movement velocity	>90% ^1^	n/a
Exercise order	Squat, Bench press, Hip thrust	Squat, Bench press, Hip thrust

^1^ Load was decreased for following set, if participant could not maintain >90% of the maximum vertical velocity.

## Data Availability

Data sharing is not applicable to this article.
